# Clinical Aspects of Adolescents Hospitalized in a Psychiatric Unit: A 12-Month Study

**DOI:** 10.1192/j.eurpsy.2025.1132

**Published:** 2025-08-26

**Authors:** A. Gharbia, D. Mezri, S. Boudriga, R. Hosni, U. Ouali, A. Aissa, R. Jomli

**Affiliations:** 1 Avicenne, Razi hospital, Mannouba, Tunisia

## Abstract

**Introduction:**

The World Health Organization (WHO) defines adolescence as the developmental stage between childhood and adulthood, spanning ages 10 to 19. This period is characterized by significant physical, cognitive, and psychosocial growth.

**Objectives:**

This study aims to understand the clinical profile of adolescents hospitalized for the first time in the psychiatric department.

**Methods:**

- It’s a retrospective and descriptive study covering the files of patients who consulted the Avicenna Psychiatry department of Razi hospital aged under 18, during the year 2023.
- The collection of anamnestic and clinical data was established using a pre-established form

**Results:**

Our study included 24 patients aged between 16 and 18 years, with a mean age of 17 years, comprising 13 males and 11 females.

Patients presented voluntarily in 42% of cases, were brought in by law enforcement in 25%, and were referred by a private psychiatrist in 17% of cases. The reasons for consultation were as follows: behavioral disturbances in 63% of cases, a suicide attempt in 21%, and incoherent statements in 8% of cases. 60% of cases used medication ingestion as the method during the suicide attempt. A family history of psychiatric disorders was found in 50% of cases, as well as a family history of suicide attempts in 13% of cases. Additionally, 71% of cases had prior follow-up with a private psychiatrist, 21%of cases had previously attempted suicide, and 33%of cases had personal somatic histories.

The onset of disturbances was progressive in 71% of patients. Upon hospitalization, anxious symptoms were noted in 25% of cases, while depressive symptoms with suicidal ideation were recorded in 29% of patients. hallucinations and delusions were noted in 50% of cases, and behavioral eccentricity was observed in 25% of cases.Instinctual disorders were present in 75% of cases, with sleep disturbances observed in 71%of cases, sexual disorders in 25%of cases, and appetite disorders in 13 %of cases.

Verbal hetero-aggression was identified in 83% of cases, while physical hetero-aggression was noted in 67%of cases. Self-harming behaviors were recorded in 48% of patients.

The diagnoses made according to DSM-5 criteria were distributed as follows: bipolar disorder in 29% of cases, schizophrenia in 17%of cases, and 13%of cases related to a life stage.

**Conclusions:**

Adolescent psychiatric hospitalizations are rising, emphasizing the need to understand clinical characteristics of this population. This study analyzes clinical aspects of adolescents admitted to a psychiatric unit over one year. The findings aim to enhance treatment strategies and patient outcomes.

**Disclosure of Interest:**

None Declared

